# A retrospective evaluation of clinical outcomes in the use of inhaled nitric oxide in acute respiratory distress syndrome caused by COVID-19: The NITRICOVID study

**DOI:** 10.5339/qmj.2026.13

**Published:** 2026-03-17

**Authors:** Mohamed Zuhail Kizhakka Peediyakkal, Muna A Rahman Al Maslamani, Nevin Kannappilly, Saifil Sidhique, Mohamed Aboukamar, Sreekanth Komath Mohan, Jintu Iype, Virendra Pratap Chaudhary, Ashib Thurakkal, Karimulla Shakeer Shaik, Solaiman Allafi, Abdulqadir Nashwan, Nabeel F Suleiman Al LObaney

**Affiliations:** 1 Hamad Medical Corporation, Hamad Medical City, Doha, Qatar *Email: mpeediyakkal2@hamad.qa

**Keywords:** ARDS, COVID-19, nitric oxide, mechanical ventilation

## Abstract

**Background:**

Acute respiratory distress syndrome (ARDS) remains a leading cause of mortality among critically ill patients with COVID-19. Inhaled nitric oxide (iNO), a selective pulmonary vasodilator, is often used as rescue therapy to improve oxygenation; however, its impact on survival remains uncertain.

**Objective:**

To evaluate the clinical outcomes of iNO therapy in patients with COVID-19-related ARDS and to stratify patients into Early, Delayed, and Non-Responder groups based on the timing of their oxygenation response.

**Methods:**

A retrospective cohort study of 99 patients with COVID-19-related ARDS who received iNO was conducted. Patients were categorized as:

**Early Responders:**

≥20% improvement in the PaO_2_/FiO_2_ ratio within 8 h;

**Delayed Responders:**

≥20% improvement in the PaO_2_/FiO_2_ ratio between 8 and 24 h;

**Non-Responders: no improvement:**

<20% improvement in the PaO_2_/FiO_2_ ratio within 24 h, including those who showed in their PaO_2_/FiO_2_ ratio within 24 h of iNO initiation.

Baseline demographics, comorbidities, and outcomes, including duration of mechanical ventilation, ICU and hospital length of stay, and mortality, were compared.

**Results:**

Early and Delayed Responders showed significant improvement in oxygenation (mean PaO_2_/FiO_2_: 137.3 vs. 126.9 vs. 106.4; *p* = 0.004), with mean percentage increases of 65.3%, 56.6%, and 8.2%, respectively (*p* < 0.001). However, this did not translate into differences in ICU mortality (64.8%, 62.5%, and 71.4%, respectively; *p* = 0.81) or other hospital outcomes. Rates of acute kidney injury (AKI), methemoglobinemia, and other complications were comparable among the groups.

**Conclusion:**

iNO improved oxygenation in a subset of patients with COVID-19-related ARDS but did not reduce mortality. Stratification by timing of response highlights patient heterogeneity and supports response-guided, time-limited use of iNO in critical care.

## 1. INTRODUCTION

The COVID-19 pandemic has imposed immense strain on healthcare systems, with acute respiratory distress syndrome (ARDS) representing one of its most lethal complications.^1^ ARDS is characterized by diffuse alveolar damage, severe hypoxemia, and high mortality rates, often necessitating invasive ventilation and rescue therapies.^2^

Inhaled nitric oxide (iNO) has long been used to improve oxygenation in severe ARDS by selectively dilating pulmonary vessels in well-ventilated lung regions, thereby enhancing ventilation–perfusion (V/Q) matching and reducing pulmonary hypertension. While it can transiently increase oxygenation indices, prior trials in non-COVID ARDS have shown inconsistent effects on mortality or duration of ventilation.^3^

The role of iNO in COVID-19–induced ARDS remains debated. Despite limited evidence, clinicians have employed it as salvage therapy in refractory hypoxemia. Observational studies have shown mixed results; some report improved oxygenation, whereas others report no benefit or possible harm.^4^ Notably, few studies have evaluated the timing of response to iNO and its relationship to outcomes. We hypothesized that distinguishing Early, Delayed, and Non-Responders could reveal clinically meaningful subgroups.

This study, therefore, assessed the effects of iNO on oxygenation and outcomes in COVID-19 ARDS, incorporating a novel stratification based on temporal response patterns.

## 2. METHODS

The NITRICOVID retrospective cohort study was conducted at a tertiary ICU and approved by the Institutional Review Board (MRC-01-24-646) between March 2020 and July 2021. Adult patients (≥18 years) with RT-PCR–confirmed COVID-19 and moderate-to-severe ARDS who received iNO were included. Exclusion criteria included non-COVID ARDS, iNO use for non-ARDS indications, pregnancy, prior lung transplant, DNR status, or incomplete records.

Ninety-nine patients met the inclusion criteria and were categorized as Early, Delayed, or Non-Responders based on oxygenation response as follows:

**Early Responders:** ≥20% rise in PaO_2_/FiO_2_ ratio within 8 h of iNO initiation.

**Delayed Responders:** ≥20% rise in PaO_2_/FiO_2_ ratio between 8 and 24 h.

**Non-Responders:** <20% rise within 24 h, including those who showed no improvement in PaO_2_/FiO_2_ ratio within 24 h of iNO initiation.

Patients received iNO per the institutional protocol, typically starting at 10 ppm (maximum 40 ppm). Continuous monitoring was performed to prevent methemoglobinemia and hypotension. Gas delivery was integrated into the ventilator circuit.

Collected data variables included:

**Demographics/comorbidities:** age, sex, diabetes, hypertension, heart disease, or kidney disease.

**Oxygenation parameters:** PaO_2_/FiO_2_ ratios pre- and post-iNO.

**Outcomes:** duration of iNO therapy, mechanical ventilation, ICU and hospital stay, incidence of acute kidney injury (AKI), need for dialysis, mortality (ICU, in-hospital, 30-day), and complications.

**Endpoints**

**Primary Endpoint:** Improvement in oxygenation (PaO_2_/FiO_2_) within 24 h of iNO initiation.

**Secondary Endpoints:** Mortality (ICU, 30-day, hospital), days on mechanical ventilation, and ICU/hospital stay.

### 2.1. Statistical analysis

Continuous variables were expressed as mean ± SD (standard deviation) or median [interquartile range (IQR)] and compared using ANOVA or Kruskal–Wallis tests; while categorical data were compared using Chi-square tests. Survival was assessed using Kaplan–Meier analysis. A *p*-value of <0.05 was considered statistically significant.

## 3. RESULTS

### 3.1. Best PaO_2_/FiO_2_ within 24 hours

“The PaO_2_/FiO_2_ (P/F) ratio represents the ratio of arterial oxygen partial pressure (PaO_2_) to the fraction of inspired oxygen (FiO_2_), expressed in mmHg.” Baseline P/F ratios did not differ significantly (*p* = 0.081). However, Early Responders achieved higher post-iNO ratios (137.3 ± 39.5) than Delayed Responders (126.9 ± 27.1) and Non-Responders (106.4 ± 29.6) (*p* = 0.004), confirming distinct oxygenation trajectories among groups.

Early Responders reached peak oxygenation fastest (mean, 6.3 h), compared with Delayed Responders (13.2 h) and Non-Responders (9.9 h), representing a highly significant difference (*p* < 0.001).

Mean P/F ratio improvement was 65.3% ± 36.2 (Early Responders), 56.6% ± 25.3 (Delayed Responders), and 8.2% ± 7.1 (Non-Responders) (*p* < 0.001), highlighting a robust physiological response in the first two groups.

### 3.2. Clinical outcomes

Despite differences in oxygenation, no significant differences were observed in major clinical outcomes (Table 1).

Mechanical ventilation duration and ICU/hospital length of stay (LOS) were similar across groups. Mortality exceeded 60% in all groups, consistent with outcomes reported in severe COVID-19 ARDS.

iNO was well tolerated. Mean methemoglobin levels remained <2% in all groups, and no major hemodynamic events were attributed to iNO. Acute kidney injury, dialysis, and infection rates did not differ significantly.

### 3.3. Long-term outcomes

Long-term complications were frequent and similar across groups. Mortality remained high (Early Responders 71.4%, Delayed Responders 62.5%, and Non-Responders 71.4%; *p* = 0.80). Among survivors, critical illness neuropathy was the most common sequela (6.1%). Rates of myopathy, stroke, and fibrosis were all <5%, with no group-wise differences, suggesting that outcomes were driven by overall illness severity rather than iNO response.

## 4. DISCUSSION

The NITRICOVID study provides a detailed evaluation of iNO use in COVID-19 ARDS and is the first to classify patients by temporal oxygenation response. Approximately two-thirds of patients demonstrated meaningful oxygenation improvement within 24 h; however, these physiological gains did not confer survival or clinical benefit.

### 4.1. Physiological insights

The significant early rise in PaO_2_/FiO_2_ among responders supports iNO’s role in optimizing pulmonary V/Q matching.^5^ Early responders (mean 6 h to peak effect) likely reflect patients with reversible pulmonary vascular dysfunction, whereas delayed improvement suggests more heterogeneous or progressive injury. Non-Responders likely had fixed shunting or extensive consolidation, limiting responsiveness.

These distinctions emphasize the heterogeneity of ARDS and may help explain the inconsistent results across prior studies that lacked temporal stratification.

Our findings mirror prior work in non-COVID ARDS. Taylor et al. (2004) and the Cochrane review by Gebistorf et al. demonstrated that while iNO transiently improves oxygenation, it does not enhance survival or reduce ventilation days.^6,7^ Similarly, a meta-analysis by Adhikari et al. found no mortality benefit, highlighting that physiological improvement alone does not equate to better outcomes.^8^

In COVID-19–specific studies, observational data have also shown transient oxygenation benefits without survival impact.^9^ Our results reinforce this pattern but add nuance by distinguishing early vs. delayed response dynamics.

### 4.2. Clinical implications

Although iNO did not affect mortality, its short-term oxygenation benefit may be clinically valuable as a bridge therapy during episodes of severe hypoxemia, particularly when other rescue measures are unavailable or contraindicated. However, the absence of long-term benefit supports a response-guided discontinuation strategy: if no ≥20% P/F improvement is observed within 8–24 h, iNO should be promptly discontinued to avoid unnecessary exposure and cost.

Consistent with previous studies, iNO was generally safe. Methemoglobin levels were low, and there were no significant differences in AKI or dialysis, aligning with recent multicenter analyses indicating minimal organ toxicity when dosed appropriately.^10^ Complication rates, such as infection and pneumomediastinum, were evenly distributed, supporting iNO’s safety in critical COVID-19 ARDS when closely monitored.

The uniformly high mortality and prevalence of critical illness neuropathy among survivors highlight the profound long-term morbidity of severe COVID-19 ARDS, irrespective of iNO response.^11^ These results suggest that outcomes are driven more by systemic inflammatory injury and prolonged critical illness than by oxygenation response alone.

The tripartite response model proposed here—Early, Delayed, and Non-Responders—offers a practical framework for individualized therapy. A key conceptual objective of the NITRICOVID study was to move beyond the conventional binary classification of “responders” and “non-responders” to iNO. We intentionally distinguished Early Responders from Delayed Responders to capture the temporal dynamics of oxygenation response, which may reflect underlying differences in pulmonary vascular reactivity, disease severity, and lung recruitability. Early improvement in PaO_2_/FiO_2_ within the first 8 h likely represents a predominantly reversible ventilation–perfusion mismatch, whereas delayed improvement may indicate more heterogeneous or evolving lung pathology requiring longer exposure to therapy. This stratification provides clinically meaningful insight by identifying patients who derive rapid physiological benefit versus those who respond more gradually, while also allowing early recognition of true non-responders, in whom continued iNO therapy may be futile. By incorporating response timing into outcome assessment, the NITRICOVID study offers a more nuanced and clinically applicable framework for guiding response-based continuation or discontinuation of iNO in severe COVID-19 ARDS.

## 5. LIMITATIONS

This single-center retrospective study carries inherent limitations, including potential confounding and selection bias. The lack of randomization limits causal inference, and unmeasured variables, such as illness severity or concomitant therapies, may have influenced outcomes. The relatively small cohort size may underpower the detection of modest outcome differences. Furthermore, long-term functional follow-up was limited.

Future prospective multicenter trials should validate response-based stratification and identify biomarkers predicting iNO responsiveness, particularly in ARDS subphenotypes with predominant pulmonary vascular pathology.

## 6. CONCLUSION

The NITRICOVID study provides a comprehensive real-world analysis of iNO use in COVID-19 ARDS. Stratification by timing of oxygenation response revealed that:

Early and Delayed Responders experienced significant short-term improvement in oxygenation (≥50% increase in P/F ratio).

Non-Responders show minimal benefit, supporting early discontinuation.

No significant differences were observed in mortality, duration of ventilation, or ICU/hospital stay.

iNO was safe, with low toxicity and stable renal outcomes across groups. Its role should therefore be considered supportive, providing transient physiological benefit without altering survival. Implementing a response-guided, time-limited approach—reassessing within 8–24 h—may optimize its clinical use.

Further, multicenter randomized studies are warranted to confirm these findings, explore predictive markers of response, and define the role of iNO in modern ARDS management strategies.

## ACKNOWLEDGMENT

The authors thank the Medical Research Center (MRC), Hamad Medical Corporation, for its support and guidance throughout the conduct of this study.

## COMPETING INTERESTS

The authors have no conflicts of interest to declare.

## AUTHOR CONTRIBUTIONS STATEMENT

MP: Conceptualization, study design, data interpretation, manuscript drafting, critical revision of the manuscript, and final approval; NK: Data acquisition, clinical data validation, statistical analysis, and manuscript editing; MA: Supervision; SS: Data extraction, data curation, and manuscript review; AK: Data acquisition, ICU clinical input, and manuscript revision; SM: Statistical analysis, methodology development, and manuscript editing. JI: Study methodology, critical review of manuscript; VC: Clinical oversight, interpretation of results, and manuscript review; AT: Data management, analysis support, and manuscript editing; KS: ICU clinical data verification and manuscript revision; AN: Study supervision, ethical oversight, and critical manuscript revision; NA: Data acquisition, literature review, and manuscript editing.

## Figures and Tables

**Table 1.** Clinical outcomes among the three responder groups to iNO therapy.

Outcome	Early responders	Delayed responders	Non responders	*P*-value	Statistical test
Best P/F ratio within 24 h of nitric oxide initiation [mean ±SD]	137.28 ± 39.45	126.92 ± 27.09	106.43 ± 29.63	0.004	ANOVA test
P/F ratio improvement % [mean ± SD]	65.35 ± 36.23	56.62 ± 25.3	8.23 ± 7.14	0.001	ANOVA test
Time to achieve best P/F ratio in hours [mean]	6.34	13.23	9.95	0.001	Kruskal–Wallis H test
Duration of mechanical ventilation in days [mean ± SD]	30.7 ± 26	30.2 ± 25.1	31.8 ± 28.5	0.94	Kruskal–Wallis H test
ICU length of stay in days [mean ± SD]	40.72 ± 32.91	37.29 ± 25.48	37.75 ± 31.01	0.812	One-way ANOVA test
Duration of hospital stay in days [mean ± SD]	53.07 ± 51.08	63.20 ± 94.09	45.25 ± 34.94	0.9	One-way ANOVA
AKI	45.50%	54.20%	50%	0.563	Chi-square test
Dialysis rates	33.33%	29.17%	33.33%	0.93	Chi-square test
Max methemoglobin levels	1.40%	1.20%	1.60%	0.216	Kruskal–Wallis H test
Respiratory infection rates after nitric oxide initiation	25.90%	25%	33.33%	0.779	Chi-square test
Nitric oxide days [mean]	5.74	7.5	9.52	0.38	Chi-square test
30-days mortality rate	27.80%	25%	38.10%	0.589	Chi-square test
ICU mortality rate	64.80%	62.50%	71.40%	0.805	Chi-square test
Hospital mortality	67.30%	62.50%	75%	0.674	Chi-square test
